# Crimes against Humanity: The Role of International Courts

**DOI:** 10.1371/journal.pone.0099064

**Published:** 2014-06-26

**Authors:** Éder Milton Schneider, José Roberto Iglesias, Karen Hallberg, Marcelo Néstor Kuperman

**Affiliations:** 1 Instituto de Física, UFRGS, Porto Alegre, RS, Brazil; 2 Programa de de Pós-Graduação em Economia, Escola de Gestão e Negócios, Unisinos, São Leopoldo, RS, Brazil; 3 Instituto Nacional de Ciência e Tecnologia de Sistemas Complexos, Rio de Janeiro, RJ, Brazil; 4 Centro Atómico Bariloche and Instituto Balseiro - CNEA, CONICET, 8400 S. C. de Bariloche, Argentina; National Scientific and Technical Research Council (CONICET), Argentina

## Abstract

We study the role of international tribunals, like the International Criminal Court (ICC), as an effective way of reducing the number and/or gravity of crimes against humanity. The action of the ICC is directed against leaders that promote or tolerate these kinds of crimes, that is, political authorities, army commanders, civil leaders, etc. In order to simulate the action of the ICC we build a hierarchical society where the most important leaders have the highest connectivity and can spread their points of view, or their orders, through a chain of less but still highly connected deputy chiefs or opinion chieftains. In this way, if they practice misconduct, corruption, or any kind of discriminatory or criminal actions against individuals or groups, it would very difficult and improbable that they will be prosecuted by the courts of their own country. It is to alleviate this situation that the ICC was created. Its mission is to process and condemn crimes against humanity though a supranational organism that can act on criminal leaders in any country. In this study, the action of the ICC is simulated by removing the corrupt leader and replacing it by a “decent” one. However, as the action of the corrupt leader could have spread among the population by the time the ICC acts, we try to determine if a unique action of the ICC is sufficient or if further actions are required, depending on the degree of deterioration of the human rights in the hypothetical country. The results evidence the positive effect of the ICC action with a relatively low number of interventions. The effect of the ICC is also compared with the action of the local national judiciary system.

## Introduction

The aftermaths of the two major war conflicts of the 20th century showed the imperative need of official institutions capable of doing justice in a global way, beyond the limits imposed by the borders of the nations. After WW1, the first initiative to judge political leaders accused of war crimes took place in the Paris Peace Conference of 1919. In this conference, the participants discussed about the convenience of establishing an international tribunal that would act when the states were absent or acted irresponsibly. At the end of WW2, two international tribunals were in charge of providing justice for the victims of the atrocities that occurred during the previous years and punished the responsible of those acts. The victorious nations established the International Military Tribunals at Nürenberg, Germany [1] and Tokyo, Japan [2]. Several years later, after serious episodes of state-sponsored crimes, similar tribunals were organized including those set up in Bosnia and Rwanda in the 1990’s. Nevertheless all these examples were isolated reactions to particular events. For example, in the case of Bosnia the United Nations created a special tribunal, the International Criminal Tribunal for the Former Yugoslavia [3]. There was a lack of a permanent and internationally recognized tool of justice to punish political leaders responsible of crimes against humanity.

A widespread consensus was achieved in Rome in 1998, where 122 countries agreed to create the International Criminal Court (ICC) as a response to the desire for international justice. The ICC was created by the Rome Statute to be a permanent, non-partisan judicial instrument to “promote the rule of law and ensure that the gravest crimes do not go unpunished [4]”. The Statute of Rome came into force in 2002 when 60 countries ratified the treaty, and the ICC was settled in The Hague, Netherlands. Its mission is to detect and judge the responsible of genocides, crimes against humanity, war crimes, and the crime of aggression. After the adoption of the Rome Statute, more than half of the countries of the world have ratified it. Since the Court was established, the Prosecutor has launched investigations into crimes committed in Uganda, Democratic Republic of Congo, Darfur (Sudan) and Central African Republic.

The main role of the ICC is to investigate and prosecute genocide, crimes against humanity and war crimes when national authorities are unable or unwilling to do so. Its actions are expected to serve as a catalyst for states to fulfill their responsibilities in investigating and prosecuting those crimes [5]. Being the ICC a fairly new institution, it has yet to show its effects as a crime deterrent [6]. In this manuscript we are not intending to judge its efficacy, instead, we aim to simulate, by means of a simple mathematical model, the role it plays in bringing justice in countries in which the authorities are not able to assume their primary obligations to warrant the citizens the necessary protection against the worst atrocities.

The interdisciplinary study of criminal activities, its economic consequences, the effect and cost of punitive actions and related subjects have been a very active area in recent years, as a natural extension of studies in opinion dynamics and game theory both in regular and complex networks [7]–[13].

Here, in order to study the role of the ICC we simulate an artificial country as a connected society with some degree of hierarchy, and the action of the ICC as a punctuated activity directed to remove evil agents in key positions. In the next section we describe the model for the artificial society and the action of the ICC while in the following sections we present the results and compare the effects of the intervention of the ICC with a possible action by national courts.

## Model

We present first a model that describesthe expansion of a criminal (or illegal) behavior as a kind of epidemics and, secondly, the corrective effect of the action of an external mechanism, such as the intervention of the International Criminal Court (ICC).

The aim of the model is to mimic a situation in which the ethical inclination of the individuals is the result of a feedback between the individual and its environment at different levels of social organization: the neighborhood, the country and the global dynamics. In turn, the attitude of each individual shapes the macroscopic behavior of the society.

A fundamental component in the study of any structurally organized criminal group is the social network [10], [14], [15]. Crimes under the ICC jurisdiction in particular are committed in the context of a hierarchical structure, and this kind of social network can be studied using complex network models [16]–[18].

**Figure 1:**
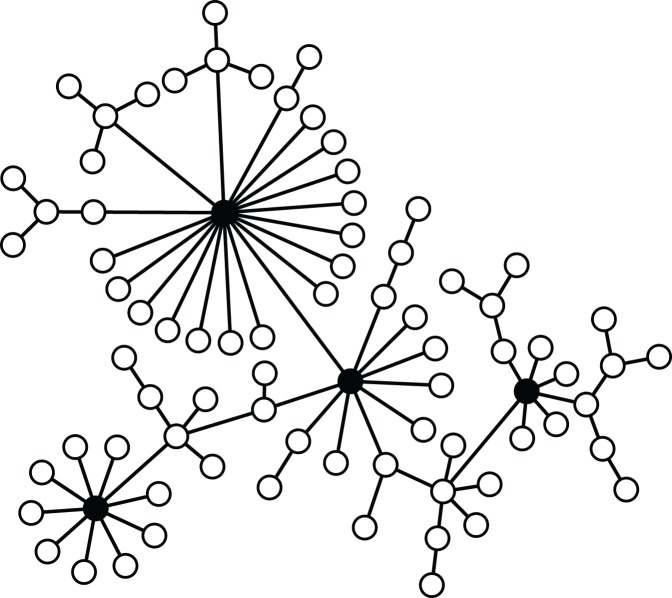
Schematic representation of a scale-free network. The “hubs”, nodes with high degree are painted black, while the low degree nodes are represented in white. Note that many nodes are related to just one hub.

The model incorporates social networks, characterized by a particular topology, as a means to represent the interactions between individuals. Each individual is inserted in a social environment socially and politically organized. This organization will be coded into the topology of a complex network, representing the underlying weave of individual connections. By means of this network we want to capture the insertion of each subject in the society. To start with, we will not consider individual connections between subjects belonging to different countries, but this interaction can be included in a subsequent analysis. By assigning different number of connections to each of the nodes of the network -the degree of the node-, the topology determines not only the neighborhood of each individual but can also indicate which type of political organization is present in the country. Consider for example that the country has a strongly democratic organization, so there will be no individuals whose decisions can absolutely outweigh those of others. Topologically, this can be translated into a hierarchical distribution with no peaks at high values. However, even in a democracy the influence of national and regional leaders is strong, the discourse of a president has a very wide network of recipients, probably more that a secretary, a governor, a major or an army general. This influence is stronger in dictatorial regimes where some kind of control of the press is also imposed. So, in different political regimes, the network of influences keeps a hierarchical structure. If one wants to consider the full details, links between individuals can also be directional, shaping the degree of influence of each of the individuals on the others. Thus, individuals are related to each other at the lowest organizational level and with many different degrees of connectivities. In order to take into account the fact that authorities, or mass media owners, or persons placed in high hierarchical positions may transmit their points of view to subordinates or people in lower hierarchical levels, we adopt a scale-free (SF) network [19] as it exhibits a wide distribution of connectivity with highly connected nodes (called “hubs”, or high degree nodes) and many nodes with very low connectivity (or low degree), forming a power law distribution of the frequency of the degree, with a connectivity exponent 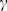. High degree nodes correspond to authorities or persons in power positions, i.e. we assume agents placed in the top of the authority and/or leadership scale will have a large connectivity, either directly or trough other mechanisms like the mass media. In Fig. 1 it is possible to see a schematic representation of the network, which exhibits highly connected nodes (painted black in the figure) linked to many nodes with one or a few connections (painted white). There exist many algorithms to create SF networks: We particularly used the Barabási-Albert (B-A) algorithm [20], in which the network is built from an initial fully connected network of 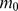 nodes by adding new nodes sequentially, each new node connecting itself to 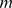 of the previous nodes with preferential attachment to the more connected ones. This network, built through growth and preferential attachment will develop into a SF network (with a power law distribution of degree exponent 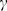) held together by highly connected hubs.

The basis of the model is the individual behavior which, as mentioned earlier, will be affected differently by the state of the components of the system in each of its levels. The behavior of each individual 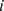 is schematically represented by a time-dependent scalar 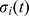 which, for simplicity, can adopt two different values, 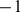 or 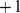, respectively representing evil and good behavior. Real situations are more subtle and less Manichean. Some individuals may adopt an evil behavior because of being afraid of retaliations, some others are concerned with the criminal behavior, but adopt a passive standing. In short, different degrees of moral behavior may be studied, as in Ref. [22], but this is a level of detail that we think would be overstated at this point of the study of the problem. So, restraining ourselves to this two state behavior, during the dynamical evolution of the system, the state of the individuals changes according to the social contacts, that we will reduce to three levels: a) The weight of the own position that will be greater the greater the influence, or connectivity, of the agent, b) The directly connected environment, i.e the influence of the agents connected to the one we are examining, and finally, c) The influence of the full society, measured as the average inclination of the agents. This last term may also represent the effect of external influences, as the international pressure applied to a given country. We remark that the method adopted here is similar to the one used to describe contagion of a disease on a complex network [11], [12]. However in our model the probability of “contagion” of the bad or good behavior will depend on the connectivity of the agent, as will be explained below. In the following we describe briefly the effect of the own state, the one of the neighbors and the average of all states on the dynamics of contagion.

### Individual Level

Each individual agent has an inner nature, either because it is pretty sure of its ideas or because it has a high adamancy, and this can prevent it from being influenced by others. textcolorblueAlso, if it is a politician it is compelled to respect certain engagements that helped it to arrive to and to keep its power position. We will consider that the weight of the own opinion is stronger the higher the connectivity, i.e. a more connected (meaning a more powerful) agent will hardly change its attitude. Mathematically we define the weight of the opinion of the 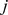-agent on itself, 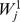 as proportional to its connectivity 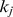:
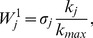
(1)where 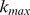 is the connectivity of the most connected agent(s). Thus, if a particular agent is the most connected one, its weight will be 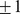, i.e. its own state. For low-connected agents one expects 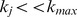, then its own opinion will have a much lesser weight.

### Local influence

Furthermore we define how the state of every agent in the social group is affected by the mood of its neighbors. We will consider as the most important influence the interaction of one agent with the other subjects directly connected to it (nearest neighbors). We define the weight of the neighbors on agent 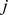 as 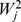. At each time step, the individual 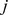 checks the state of its neighbors and makes a weighted average of these states to get a value between −1 and 1, calculated as
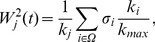
(2)where 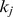 is the degree of agent 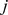, 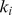 is the degree (connectivity) of the 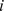-neighbor, 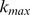 is the maximum degree present in the network, and 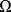 denotes the full neighborhood of a node. Here, we have assumed that the degree of a node, i.e. the size of its neighborhood, is a measure of its social influence. Thus, agents with the highest degree will exert a stronger influence on their neighbors. On the other hand, the influence of the neighbors on agent 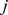 is divided by its own connectivity 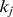. The higher 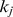 the lower the weight of the opinion of the neighbors.

### Global Level

While the individual level defines a type of local influence, the global level defines the environmental effect in a wider range. This level takes into account the topology of the influences of the whole country to weigh the influence of each of the individuals over the rest, independently of whether they are connected or not. We collect the information already considered at the individual level but use it in a wider sense. We calculated a weighted average of the state of each of the individuals in the country to get 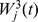, the weight of the global influence on agent 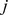.
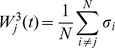
(3)where 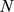 is the number of individuals in the country. This term is an average of the national opinion, however it is probably the less effective in changing the state of one agent.

We remark that the dynamics of contagion is different from previous studies on the diffusion of a disease in a complex network [12]. The agents are not equivalent in their resistance to adopt a different moral standing: Hubs are almost insensitive to their environment while less connected agents are more susceptible. In societies where crimes against humanity are detected other factors are relevant to the adoption of an immoral attitude. Cultural and historical factors are also relevant. However, as we are more interested here in the ICC action than on the details of how a society could eventually endorse genocides or other crimes against humanity, we skip the details of the corruption process and retain the importance of agents in high hierarchical position and the consequences of their possible eradication.

One can further include an international influence, by taking into account interactions with other countries. Again, we can consider local or global interactions. The bilateral relations between countries can be also represented by a network of communities, analogously to what we have done with individual interactions or individual national levels. We should define a topology for the network of countries and consider that countries are new individuals that interact with each other in the same way agents did in individual and local levels. However, in order to isolate the effect of each of the terms considered, and also to minimize the number of parameters in the model, in this first stage we will consider just a single country and do not include the international contacts.

## Results

We consider two stages in the dynamics: a) Contagion effects: This is the change in the state of the individuals of the system as a result of the different influences described above. The contagion may in some cases induce a state of complete polarization in the evil or good state. b) Action of the ICC. The action of the ICC will be sporadic and will be directed to remove from the society an evil hub, i.e. a highly connected agent that exerts an evil influence on the community. We can also say that the contagion effect is internal to the society, while the action of the ICC is external. Hereafter, we are going to discuss both dynamics separately.

### Internal dynamics: Contagion

**Figure 2:**
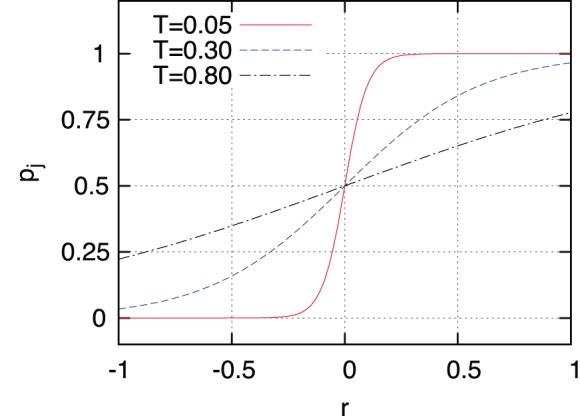
Probability of changing state for different social “temperatures”. Probability of changing state to state 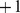 as a function of 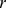 for different values of the “social” temperature, given by Eq. 5. For high temperatures the probability is almost linear in 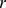 while for low temperatures it approaches a step function.

Let’s consider that the three influences described above have different weights, being the first two, the own and local influences, the stronger, and the national one, the weaker. If we increase too much the national influence the network effects are erased and a kind of mean-field solution prevails. For each agent 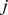 we calculate a change exponent 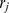 defined as:
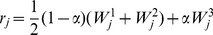
(4)where the parameter 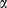 is in the range 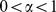 and measures the relative weight of the effect of the national level, compared with the individual and local ones. A typical value is 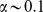 (bigger values of 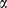 will increase the effect of the average opinion and erase the influence of the network; the final state will depend only on which of the attitudes is preponderant at the beginning). It is evident that each of the terms in Eq. 4 may be positive or negative and then 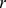 itself maybe positive or negative. One possible dynamical evolution would be to prescribe that if 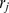 is negative (positive) at a given time step, agent 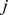 will remain evil (good) or change to the evil (good) state 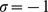 (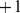). To avoid such a deterministic behavior we introduce a factor of “social” temperature (that is: a non-zero probability of spontaneous changes) in the probability of changing attitude. This temperature takes into account spontaneous and unpredictable changes of attitude, but also the fact that in some situations fractions or the whole population can exhibit a certain alienation, that is, a confusion about what right or wrong is. So, we define a probability 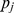 that agent j changes to (or remains in) the state 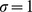 as:
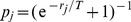
(5)where 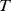 is the social temperature. One can observe that if 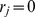, 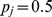, then agent 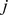 can adopt either of the attitudes with equal probability. If 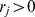 (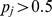) there is a higher probability of changing to the good state, while if 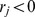 (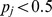) there is a higher probability of changing to the evil state. We have represented in Fig. 2 the probability of change as a function of 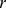 for different values of the social temperature. We assume a low value of the national influence, by considering 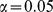. We will keep this value all along the simulations, in order to emphasize network effects over ambiance effects.

**Figure 3:**
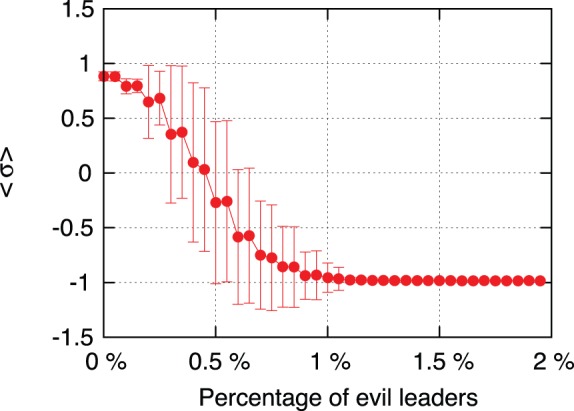
Effect of the initial number of evil leaders. The figure shows the final state of the society as a function of the initial fraction of evil leaders without ICC intervention. One may see that a small percentage of the population (less than 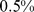) occupying the most connected positions, may transform the full society to the evil state. We have considered 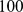 samples with 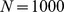 agents each, solid lines describe the averaged values over all the simulations, and the error bars correspond to the standard deviation. Note that the transition region with high error bars shows that the final state of the different samples may be a completely degraded or non-degraded populations.

Now, in order to study the dynamics of contagion, we assume a network with 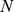 agents (in the simulations we will consider 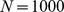 and 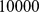 but, unless explicitly stated, we always present the results for 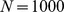, as they are similar for larger systems). Also, we make the hypothesis that almost the full society is initially in the good state (in average 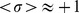). However, there will be a very small fraction of evil agents placed in sites with high connectivity that will trigger the contagion. To show this point we have made a simulation of a SF network using the B-A algorithm [20] with 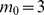 initial nodes, 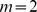 nodes added each growth step and 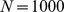 agents at the end (which gives a power law exponent of about 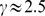), placing a small percentage of evil agents in the most connected positions. Also, we have performed an average over 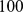 samples. The deleterious effect of the strategically placed evil agents can be seen in the simulations plotted on Fig. 3, where one can verify that a very small percentage of evil agents (about 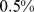) in key positions (hubs) is enough to drive the full society to an evil behavior (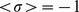). As the value of 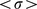 determines the behavior of the society (good if 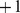, evil if 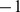) we will call it the *moral* index.

So, it is clear that evil agents placed in strategic positions of high hierarchy or authority may induce a despicable behavior of the whole population in a very short lapse of time.

### Action of the ICC

We model now the regulatory effect of an international institution acting on individuals, penalizing those whose behavior falls under the guise of “crimes against humanity”. In this sense the simulation imitates the objectives of the ICC: Bringing to the Court the highest accountable leaders such as presidents, prime ministers, army commanders or others. In our model, the action of the Court is simulated by removing the highest connected evil leader. Because of the length of the investigations, the action of the court can take place with some delay, and during the delay, the evil behavior spreads and can affect the whole society (contagion effect). The removal of a high-degree node from the lattice may produce undesirable effects and even destroy the unity of the network [21]. Also, if the removed authority is substituted by a new one, the connections of this newcomer maybe very different of the original ones. Consequently, the aftereffects of the demotion of a leader maybe very difficult. In this first approach to the problem we have decided to avoid the complications arose by the re-designing of the network and we have considered that the removed evil leader is just replace by a “good” one. This is a simple approach but we believe it contains the essential of the action of the Court, particularly because, after the contagion, the new leader will be surrounded by lots of evil agents as well in the old or in a new structure of neighborhood.

Thus, the simulations proceed in the following way: We build a SF network with the B-A algorithm (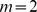, 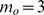 and 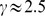). The number of agents is initially taken to be 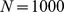 and averages of 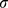 over 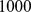 samples are studied. The global factor in the calculation of the probability of changing mood is 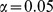 and a percentage of 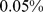 initial evil leaders is placed in the most connected hubs. Finally, the social temperature is chosen to be 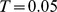 in order to obtain a society with a well defined polarization. The Court acts periodically, every 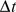 contagions steps, replacing the most connected evil agent by a good agent and in between the system evolves with its own contagion dynamics.

**Figure 4:**
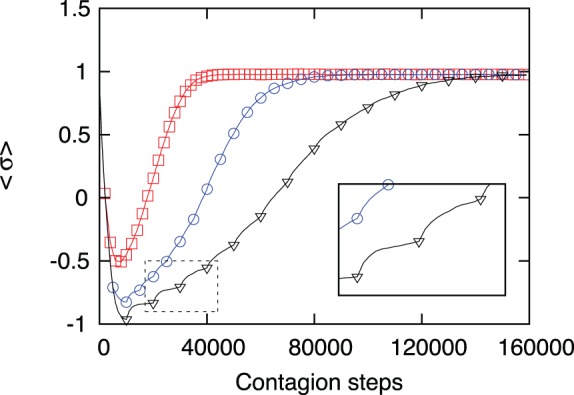
Moral index of the society as a function of time. The figure shows the evolution of the moral indexs when contagion is combined with the action of the ICC. Three values of the time separation among ICC’s interventions are shown: 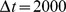 (red squares), 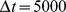 (blue circles) and 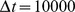 (black triangles). Early intervention (after 2000 time steps) prevents the contagion of the full society and the recovery is relatively rapid. Longer times for interventions (5000 or 10000 time steps) result in a deteriorated society and longer recovery times. In these simulations we use 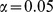, 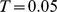 and an initial fraction of 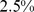 evil leaders and we average over 1000 samples. The points along the curves correspond to ICC interventions and are not the only data points drawn, the curves between ICC actions are composed by one data point per step. Looking in greater detail the dotted box it’s possible to see a change in the derivative: i.e. a quick increase in the moral index following each intervention of the Court; This rapid but small increase corresponds to the local influence of the new good agent replacing the evil leader removed.

**Figure 5:**
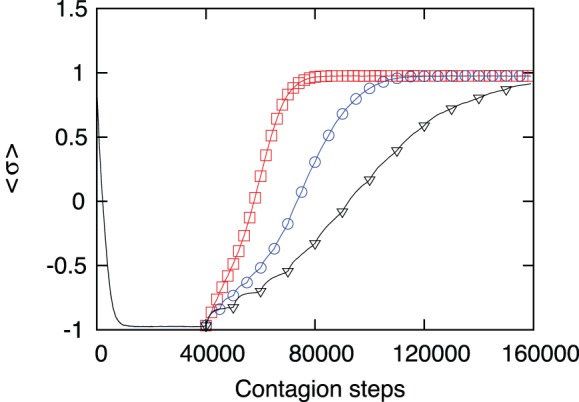
Recovery of the moral index of the society after full deterioration. If one waits 40000 time steps for the action of the Court, the society is fully degraded when the Court finally acts. However the time to full recovery is not very different from the previous situation. Here the Court acts after 40000 time steps and after that the action is repeated after 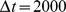 (red squares), 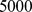 (blue circles) or 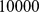 (black triangles) time steps. This simulation used 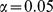, 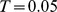 and a percentage of 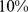 evil leaders; results are averaged over 1000 samples.

The following figures represent different situations: In Fig. 4 we show the evolution of the average behavior of the society. An early intervention of the ICC guarantees a rapid and full recovery. However, this is probably unrealistic, as the detection of violations of human rights is not an easy and rapid task. To simulate a more realistic situation, in Fig. 5 we show the time evolution after the full deterioration of the moral index. It is comforting that even in this case, when the action of the ICC starts from 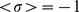, the recovery is complete but, as expected, within a longer recovery time.

### Efficiency and Relevance of the ICC

After verifying the effect of the intervention of the ICC some questions may arise concerning the real necessity of this international court. Opponents to this mechanism may argue that national courts are able to do the job if allowed for and that there is no need of an international intervention. To address this issue we will compare the action of the ICC with the possible intervention of national courts in an environment where human rights and basic law principles are violated.

#### Can national courts do the work of the ICC?

**Figure 6:**
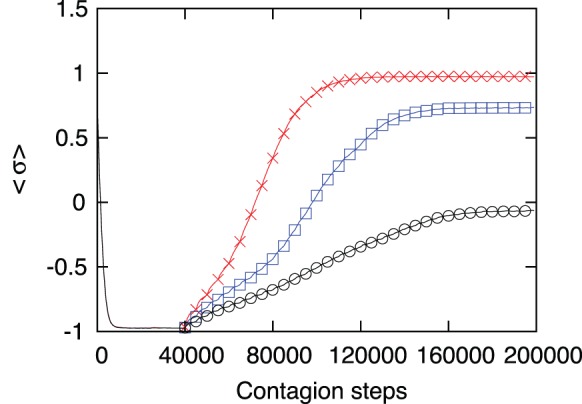
Comparison of the action of the ICC vs. national courts. The moral index of the society is represented as a function of time, comparing the action of the ICC (red crosses) every 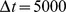 times, with the action of national courts protecting the top 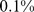 (blue squares) or 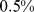 (black circles) evil leaders. This simulation used 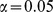, 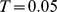 and a percentage of 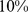 evil leaders, results are averaged over 1000 executions.

**Figure 7:**
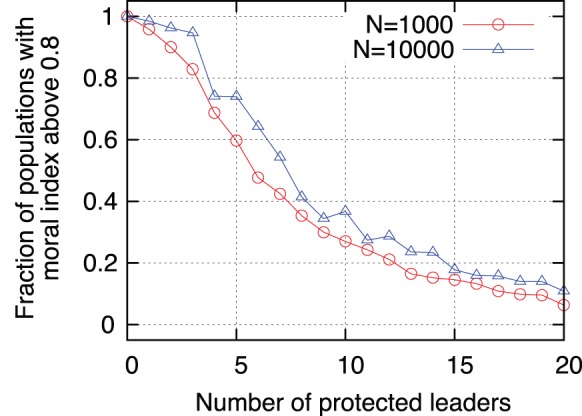
Society recovery as a function of the fraction of “protected” agents. Fraction of the simulated society that recovered up to a moral index higher than 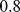 as a function of the number of agents “protected” by national rules (or by corruption) from the prosecution of national courts. Red circles correspond to a sample of 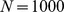 agents and blue triangles to a society of 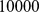 agents. This simulations used 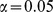, 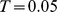 and a percentage of 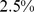 evil leaders; results are averaged over 100 (red circles) and 30 (blue triangles) executions.

It is evident that a national court taking the same measures as the ICC would produce the same effect. However the problem with national courts is that, even when they act within the law, frequently they are not able to process highly ranked officials. Quite often, the law, the practice or the custom is such that highly placed officials cannot be subjected to prosecution, and only intermediate or low level officials can be prosecuted. Thus, in order to compare the effect of the action of national courts, we have repeated the simulations of the previous section but, instead of prosecuting the most connected agent we have selected highly but less connected agents. Thus, we made a simulation similar to the one shown on Fig. 5, with intervention of the national courts every 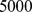 time steps, but assuming that the national court cannot prosecute the most connected agents. The results are shown in Fig. 6 where it is evident that the recovery of the society is faster and complete when the most connected leaders are subjected to process than when a fraction of the leaders is protected from prosecution. To better illustrate this effect, in Fig. 7 we show the fraction of times the society recovers up to a moral index higher than 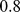 as a function of the number of agents that are screened from prosecution because of national laws or due to corruption. It is evident that the prosecution of the most connected leaders is necessary for a full social recovery. When a fraction of the leaders is protected from prosecution the recovery of the society is only partial and will degrade again when the court interrupts its action. One interesting result is that the absolute number of protected agents necessary to produce a given level of recovery is independent of the size of the society. In Fig. 7 we present results for a society of 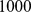 and 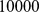 agents and the curves are very similar, indicating that one needs to prosecute a low number of agents and not a percentage (that would be impossible in a society of millions of people). This is a validation of the model and of the simulation, showing that there is no need to perform simulations with millions of agents. As a matter of fact, even if sometimes a big fraction of the society is concerned with human rights violations, most of the time the blamable agents are authorities or highly placed leaders and their number maybe well estimated in the thousands.

#### How many times should the ICC intervene?

**Figure 8:**
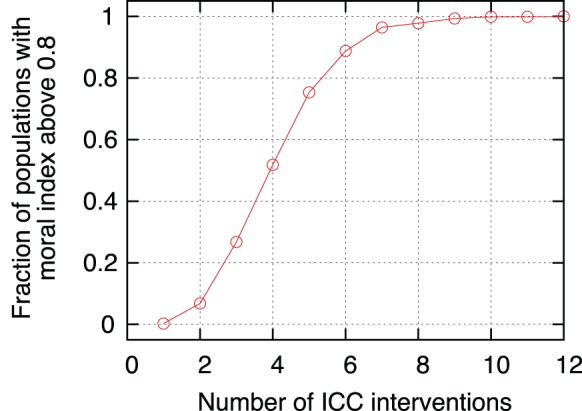
Minimum number of actions of the ICC required. Fraction of the simulated societies that recovered up to a moral index higher than 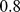 after a number of interventions of the ICC. This simulation used 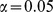, 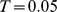 and a percentage of 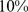 evil leaders, results are averaged over 100 executions. The interval 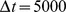 between CPI actions is used but the same result is obtained for other values of 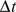, as long as the total amount of steps of each simulation is sufficient for the system to stabilize once the actions have ceased. In this figure a total amount of 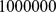 steps is used.

Once we have shown that the effect of the ICC is different and more efficient than the work of national courts, one could ask how many interventions of the ICC are necessary to induce full recovery of a given society. In the previous sections we have simulated periodic interventions of the ICC. The question is whether it is really necessary to act periodically or if just a finite number of actions are enough to produce the desired effect. To answer this question we show in Fig. 8 the number of times a simulated society arrives to moral index of 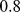 or higher (i.e. 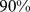 or above of the population in the “good” state) as a function of the number of actions of the ICC. It is a very good sign that a small number of interventions, of the order of five, is enough to guarantee recuperation of most of the simulated societies. Probably a more realistic simulation, including the effects of a rebuilding of the network, will reduce even more the minimum number of interventions.

### Conclusions

We have presented here a simple model of a scale free complex network representing the network of influences in a society with a hierarchical structure. The state of each member of this society is represented, in a rather Manichaean way, as good or evil. To start the contagion dynamics we plant a very few bad seeds in key positions, i.e. highly connected hubs. One of the original features of this article is that the probability of contagion strongly depends on the connectivity: Hubs are excellent transmitters of behavior and very resistant to changes induced by their neighbors. On the other hand agents with one or a few connections are very susceptible to contagion. This is at variance with most models of contagion on complex networks, where the most connected agents are more susceptible to infection. Once the society is partially or fully contaminated with the bad behavior we simulate the action of the International Criminal Court, ICC, by removing the most connected bad agent and replacing it by a good one. The fact that this new good agent possess a great capacity of contagion, implies that a few interventions of the ICC allow for the complete recovery of the society. We have proven also that the action of the ICC is different and much more effective than the one of national courts. The model still needs some improvements. We need to incorporate a more detailed description of the topology of the social network, including dynamical networks as well as different architectures to mimic a wider spectra of political regimes. Also, the influence of neighboring countries should be considered. Thus, in future developments we plan to simulate a dynamic network and, in further stages interaction between different countries, showing particularly that impunity is an incentive to the ethic degradation of countries, just because of imitation. Nevertheless, the present results are very encouraging, as they show that the strategy of attacking the top corrupt figures in a political system is the best suited to diminish the number of crimes against humanity and genocides. It is interesting to note that the fact that the leaders deserve great visibility facilitates the actions of the courts. This is not the case of organized crime, where the leaders are frequently invisible. If in the near future more countries will adhere to the ICC statute, the action of the Court will be even more effective.
